# Phage Display Selection, Identification, and Characterization of Novel Pancreatic Cancer Targeting Peptides

**DOI:** 10.3390/biom10050714

**Published:** 2020-05-05

**Authors:** Mallika C. Asar, April Franco, Mette Soendergaard

**Affiliations:** 1 Department of Chemistry, Western Illinois University, 1 University Circle, Macomb, IL 61455, USA; m-asar@wiu.edu; 2 Department of Biological Sciences, Western Illinois University, 1 University Circle, Macomb, IL 61455, USA; a-franco@wiu.edu

**Keywords:** pancreatic cancer, phage display, peptides, phage, cancer targeting

## Abstract

Pancreatic cancer is characterized by a 5-year survival rate of 3%, in part due to inadequate detection methods. The small size of peptides offers advantages regarding molecular targeting. Thus, peptides may be used in detection of pancreatic cancer. Here, peptides that target pancreatic cancer cells were selected using phage display technology using a 15-mer fUSE5 library. Phage were pre-cleared against immortalized pancreatic cells (hTERT-HPNE), followed by selections against pancreatic cancer (Mia Paca-2) cells. Next-generation sequencing identified two peptides, MCA1 and MCA2, with a Log2 fold change (Mia Paca-2/ hTERT-HPNE) >1.5. Modified ELISA and fluorescent microscopy showed that both peptides bound significantly higher to Mia Paca-2 cells, and not to hTERT-HPNE, embryonic kidney (HEK 293), ovarian (SKOV-3) and prostate cancer (LNCaP) cell lines. Further characterization of MCA1 and MCA2 revealed EC_50_ values of 16.11 µM (95% CI [9.69, 26.31 µM]) and 97.01 µM (95% CI [58.64, 166.30 µM]), respectively. Based on these results, MCA1 was selected for further studies. A competitive dose response assay demonstrated specific binding and an IC_50_ value of 2.15 µM (95% CI [1.28, 3.62 µM]). Taken together, this study suggests that MCA1 may be used as a pancreatic cancer targeting ligand for detection of the disease.

## 1. Introduction

Pancreatic ductal adenocarcinoma is currently the fourth leading cause of cancer-related deaths in the United States [1]. One of the main hallmarks of pancreatic cancer is rapid tumor invasion and systemic dissemination throughout the body [2]. The aggressive metastatic progression combined with asymptomatic disease development and inadequate diagnostic methods lead to an average 5-year survival rate of approximately 3% [1,3]. Current diagnostic methods include computed tomography (CT) and endoscopies; however, these technologies often lead to false-positive diagnoses, or fail to detect small tumors (<10 mm) [4,5,6]. Measurement of the levels of the serum carbohydrate (or cancer) antigen 19-9 (CA19-9) is currently in use for detection of pancreatic cancer [7,8,9]. However, this method is inadequate for early diagnosis due to low expression levels. Additionally, CA19-9 levels have been associated with other gastrointestinal malignancies, as well as benign pancreaticobiliary diseases such as pancreatitis and cholangitis [10]. Thus, improved detection of pancreatic cancer is needed to enhance prognosis for patients.

Bacteriophage (phage) display technology employs the use of libraries containing up to 10^9^ different virions that are genetically modified to express foreign polypeptides [11,12,13,14]. Peptide phage display has been used to select ligands that specifically bind cancer cells and that have been used to target tumors in vivo for diagnostic purposes [15,16,17,18,19,20,21,22]. Peptides are advantageous as diagnostic agents due to their rapid clearance from the blood, increased tissue penetration, non-immunogenic nature, and easy synthesis [23,24].

Phage display selections may be carried out against isolated cancer biomarkers and other molecules (selector) that are known to be upregulated in malignant cells. One such example includes the KCCYSL peptide that was selected against the human ErbB2 (HER2/Neu) receptor [25] and subsequently used in imaging of ovarian and breast cancer tumors in mice [18,26]. Additionally, peptides that bind the carcinoma-associated carbohydrate Thomsen–Friedenreich (TF) antigen were selected using isolated glycoproteins [27]. One of these peptides was later used in positron emission tomography (PET) imaging of breast tumors in mice [28]. Although many peptides selected against isolated molecules have been successfully employed in in vivo targeting, several have later been found to bind the tumor vasculature [29,30]. However, “blinded” selections that use whole cells or tissues, and where the molecular selector is unknown, have resulted in peptides with excellent imaging capabilities [16,17,22]. Specifically, a two-tier in vivo/in vitro phage display selection against ovarian adenocarcinoma (SKOV-3) cells identified peptide J18 (RSLWSDFYASASRGP) that has been used in single photon emission computed tomography (SPECT) imaging of xenografted ovarian tumors in mice [17]. While the molecular target of peptide J18 is unknown, the peptide exhibited excellent imaging properties with minimal background binding [17].

Here, a “blinded” phage display selection using a fUSE5 15-mer peptide library that was pre-cleared in nude mice [17] was carried out to identify peptides that target pancreatic cancer cells (Mia Paca-2). Two peptides (MCA1 and MCA2) were selected and evaluated regarding their cell specificity, binding affinity (half-maximal effective concentration; EC_50_), and binding specificity (half maximal inhibitory concentration; IC_50_).

## 2. Materials and Methods

### 2.1. Chemicals and Reagents

Unless otherwise noted, all reagents in this study were purchased from Thermo Fisher Scientific (Waltham, MA, USA).

### 2.2. Cell Lines

The human cell lines were purchased from American Type Tissue Culture (ATCC, Manassas, VA, USA) and maintained according to the instructions. Normal immortalized pancreatic cells (hTERT-HPNE) were propagated in 75% Dulbecco’s Modified Eagle Medium (DMEM) with 2 mM L-glutamine, 1.5 g/L sodium bicarbonate, 25% M-3 base medium, (Incell Corp, San Antonio, TX, USA), 5% fetal bovine serum (FBS), 10 ng/mL human recombinant epidermal growth factor (EGF), 5.5 mM glucose, and 750 ng/mL puromycin. Pancreatic cancer cells (Mia Paca-2) were propagated in DMEM supplemented with 10% FBS, and 5% horse serum. Ovarian cancer cells (SKOV-3) were grown in McCoy’s 5A supplemented with 10% FBS. Prostate cancer cells (LNCaP) were propagated in RPMI 1640 supplemented with 10% FBS. Human embryonic kidney cells (HEK 293) were grown in DMEM supplemented with 10% FBS. All media were supplemented with 50 µg/mL gentamicin, and all cell lines were maintained at 37 °C, and 5% CO_2_.

### 2.3. Phage Display Selection

For selection of pancreatic cancer targeting peptides, a 15-mer fUSE5 phage display library (a kind gift from Dr. George Smith [11,31]) was used. Amplification of the library in *E. coli* K91BK and isolation using polyethylene glycol (PEG)/NaCl precipitation were done as previously described [32]. The concentration of phage particles (virions per mL; V/mL) was measured spectrophotometrically at 269 nm and 320 nm, as described previously (SPECTRAMAX 250, Molecular Devices, Hampton, NH, USA) [32].

The library was previously pre-cleared in nude mice [17], according to Newton et al., to remove phage that bind to the vasculature and non-target tissues [33]. For negative selections, approximately 10^6^ normal pancreatic hTERT-HPNE cells were washed with 5 mL ice-cold phosphate buffered saline (PBS), and then incubated with 5 × 10^13^ V of pre-cleared fUSE5 15-mer library for 1 h at 30 rpm and 4 °C. The unbound phages were collected by aspiration, and bound phages were eluted using 2.5% 3-cholamidopropyl dimethylammonio 1-propanesulfonate (CHAPS). Unbound and bound phage were separately amplified in *E. coli* K91BK and isolated by (PEG)/NaCl precipitation [32].

For positive selections, approximately 10^6^ Mia Paca-2 cells were washed with 5 mL ice-cold PBS, and incubated with 10^13^ V of the amplified unbound phage (negative selection) for 1 h at 30 rpm and 4 °C. Unbound phage were collected by aspiration, and the cells were washed three times with ice-cold tris-buffered saline (TBS). The bound phages were eluted by 2.5% CHAPS, and then amplified and isolated as described above. The positive selection process was repeated as described three more times. In the fourth and final round, approximately 0.5 × 10^6^ cells were used to increase the stringency of the selection.

### 2.4. Next-Generation Sequencing and Bioinformatic Analysis

For identification of selected peptide sequences, phage DNA was analyzed by next-generation sequencing. The concentration and purity of isolated phage DNA and polymerase chain reaction (PCR) amplicons were determined spectrophotometrically at 260 nm and 280 nm (NanoDrop 2000, ThermoFisher Scientific, Waltham, MA, USA).

Phage from the negative selection (bound fraction) and the last round of positive selection were amplified in *E. coli* K91BK as described above, and the phage single-stranded DNA was isolated using the HiSpeed Plasmid Midi Kit (Qiagen, Hilden, Germany). The foreign nucleotide sequence encoding the displayed peptide was then amplified using PCR. The reaction included 2.5 µL fUSE5 (10 µM) primers (forward primer: 5′-ACTCGGCCGACGGGG-3′; reverse primer: 5′-TTTCAACAGTTTCGGCCCCA-3′), 2 µL isolated phage DNA, 18 µL nuclease free water, and 25 µL USB Fidelitaq PCR Master Mix. The step program used was as follows: initial denaturation at 94 °C for 2 min, denaturation at 94 °C for 30 s, annealing at 62 °C for 30 s, extension at 68 °C for 2 min, and final extension at 68 °C for 5 min. The QIAquick PCR Purification Kit (Qiagen, Hilden, Germany) was used to purify the PCR amplicons, which were then analyzed by next-generation sequencing (Genewiz, South Plainfield, NJ, USA) on an Illumina MiSeq (Illumina, San Dieso, CA, USA) using an 2 × 150 bp configuration. Paired-end reads were merged into a single sequence if they overlapped. Unique nucleotide sequences were identified, and their abundances were calculated. The unique nucleotide sequences were translated to amino acid sequences and their abundances were calculated. The Log2 (fold change) was calculated for the most abundant clones (positive selection/negative selection). Clones with a Log2 > 1.5 were selected for further studies. The top 10 peptide sequences, as identified by next-generation sequencing, were analyzed using the NCBI- Basic Local Alignment Search Tool (BLAST), PepBank database, and the Google search engine to identify sequence similarities to known peptides or proteins.

### 2.5. Peptides

Biotinylated peptides MCA1, MCA2, and J18 (SKOV-3 targeting control; [17]) were synthesized with a biotin group and an amino acid glycine–serine–glycine (GSG)-spacer at the N-terminus and an amide at the C-terminus (biotin-GSG-MCA1, biotin-GSG-MCA2, biotin-GSG-J18). Peptides (unbiotinylated) MCA1 and MCA2 were synthesized with a GSG-spacer at the N-terminus and an amide at the C-terminus (GSG-MCA1 and GSG-MCA2). All peptides were synthesized by Genscript (Piscataway, NJ, USA).

### 2.6. Fluorescent Microscopy

Mia Paca-2, hTERT-HPNE, SKOV-3, LNCaP, and HEK 293 cells were grown to approximately 80% confluency on 8-well chamber slides (Nest Scientific, Rahway, NJ, USA). The cells were incubated with 10 µM biotin-GSG-MCA1, biotin-GSG-MCA2, biotin-GSG-J18, or dimethyl sulfoxide (DMSO; vehicle) for 1 h at 37 °C and 5% CO_2_. The cells were washed with PBS and then fixed using 10% formalin for 10 min. Next, the cells were washed thrice with PBS and blocked using 10% FBS, 0.3 M glycine, 0.05% Tween-20 in PBS for 1 h. The cells were then probed by streptavidin–fluorescein isothiocyanate (FITC) for 1 h, and the cells were washed with 0.05% Tween-20 in PBS. The slides were mounted using Pro Longwear Anti-Fade Mountant with 4′,6-diamidino-2-phenylindole (DAPI) and left to cure overnight in the dark. Bound peptides were visualized using an epifluorescent EVOS FLoid Cell Imaging Station (ThermoFisher Scientific, Waltham, MA, USA). The fluorescent intensity per cell was quantified by calculating the corrected total cell fluorescence (CTCF) using ImageJ software (v1.52a, National Institute of Health, Bethesda, MD, USA) [34]. The mean background (non-cell) fluorescent intensity was used to adjust the mean fluorescent intensity per cell (region of interest; ROI).

### 2.7. Modified ELISA

The binding and cell specificity of the selected peptides to Mia Paca-2, hTERT-HPNE, SKOV-3, LNCaP, and HEK 293 cells were further evaluated using a modified enzyme-linked immunosorbent assay (ELISA), as previously described [16]. In brief, cells were incubated with 10 µM biotin-GSG-MCA1, biotin-GSG-MCA2, biotin-GSG-J18, or DMSO (vehicle) for 1 h at 37 °C and 5% CO_2_. The cells were then washed with 1% bovine serum albumin (BSA) in PBS and fixed using 10% formalin for 10 min. Following extensive washing with 1% BSA/PBS, the cells were blocked using 10% FBS, 0.3 M glycine, 0.05% Tween-20 in PBS for 30 min. The bound peptides were then probed by horseradish peroxidase (HRP)-conjugated streptavidin for 1 h, after which the cells were washed with 0.05% Tween-20 in PBS. Bound peptides were detected spectrophotometrically at 405 nm after addition of the HRP substrate 2,2’-azino-bis(3-ethylbenzothiazoline-6-sulfonic acid (ABTS; SPECTRAMAX 250 microplate reader, Molecular Devices, Hampton, NH, USA).

The modified ELISA was further used to determine the half-maximal effective concentration (EC_50_) of peptides biotin-GSG-MCA1 and biotin-GSG-MCA2 for Mia Paca-2, hTERT-HPNE, SKOV-3, LNCaP, and HEK 293 cells. The assay was performed as described above, except each cell line was incubated with 0.1–300 μM biotin-GSG-MCA1 or biotin-GSG-MCA2 to assess the dose–response relationship. Following the 1 h incubation at 37 °C, 5% CO_2_, the cells were washed (1% BSA/PBS), fixed (10% formalin), and blocked (10% FBS, 0.3 M glycine, and 0.05% Tween-20/PBS). Peptide binding was detected spectrophotometrically (405 nm) using HRP-streptavidin and ABTS (SPECTRAMAX 250, Molecular Devices, Hampton, NH). The data were fitted to a dose–response (stimulation) curve using non-linear regression (GraphPad Prism vs. 8.3.0, GraphPad Software Inc, San Diego, CA, USA).

The specific binding and the half-maximal inhibitory concentration (IC_50_) of biotin-GSG-MCA1 peptide were evaluated using a competitive modified ELISA. For this, the biotinylated peptides were employed in competition with increasing concentrations of the unbiotinylated peptides for binding to Mia Paca-2 cells. The cells were preincubated with various concentrations (0.03–100 μM) of unbiotinylated peptide GSG-MCA1 for 15 min at 37 °C, 5% CO_2_ after which 30 μM biotin-GSG-MCA1 was added for 1 h at 37 °C, 5% CO_2_. After washing (1% BSA/PBS), the cells were fixed by 10% formalin for 10 min, and extensively washed using 1% BSA in PBS. Cells were then blocked by 10% FBS, 0.3 M glycine, and 0.05% Tween-20 in PBS for 30 min. Horseradish peroxidase-conjugated streptavidin was then added for 1 h and the cells were washed with 0.05% Tween-20 in PBS followed by the addition of ABTS. Lastly, the absorbance at 405 nm was measured spectrophotometrically (SPECTRAMAX 250, Molecular Devices, Hampton, NH). The data were fitted to a dose–response (inhibition) curve using non-linear regression (GraphPad Prism vs. 8.3.0, GraphPad Software Inc, San Diego, CA, USA).

### 2.8. Statistical Analysis

Statistical analysis was done to determine significance using a one-tailed unpaired *t*-test (GraphPad Prism vs. 8.3.0). A *p*-value of <0.05 was considered significant.

## 3. Results

Phage display selection using a fUSE5 15-mer library was performed to identify peptides that specifically bind to human pancreatic cancer cells. The phage display library was previously pre-cleared in nude mice to remove non-cancer targeting clones [17].

### 3.1. Phage Display Selection and Next Generation Sequencing

In this study, the library was further subjected to a negative selection round against immortalized human pancreatic cells (hTERT-HPNE), which was followed by four rounds of positive selections against human pancreatic cancer cells (Mia Paca-2). Finally, selected phage-displayed peptides were identified by next-generation sequencing of PCR amplicons corresponding to the foreign nucleotide insert. The results identified 102,145 and 31,748 different phage clones in the negative and fourth positive selections, respectively. Further bioinformatic analyses sorted the sequences according to abundance, which is an indicator of target affinity. Thus, the Log2 (fold change; positive/negative selection) was calculated for the 10 most abundant clones in the positive selection. These 10 clones were present in both the positive and negative selections. The 10 peptide sequences were analyzed using the NCBI-BLAST and PepBank databases, as well as the Google search engine to identify sequence similarities to known peptides or proteins. The results showed that MCA3 was previously identified in phage display selections against caveolin [35], and mouse zona pellucida [36], and was used as a negative control peptide in studies targeting cancer antigens [37,38]. Peptide MCA4 was previously identified in selections against ovarian cancer [17], prostate cancer cells [33], and was used as a negative control of a malarial antigen [39]. For this reason, MCA3 and MCA4 were eliminated from further studies. Given that MCA1 and MCA2 have not previously been identified and are the only clones with Log2 values above the cutoff of 1.5, these two phage clones were selected for further study (Table 1).

### 3.2. Fluorescent Microscopy

**Figure 1:**
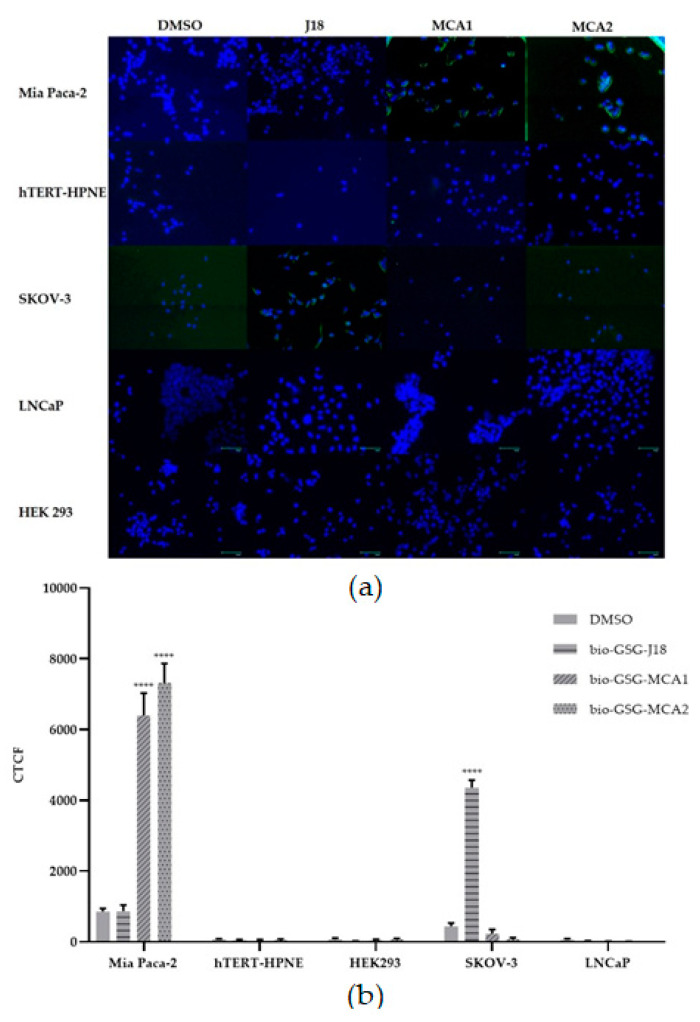
(**a**) Fluorescent microscopy of peptides biotin-GSG-MCA1, biotin-GSG MCA2, or biotin-GSG J18 to Mia Paca-2, hTERT-HPNE, SKOV-3, LNCaP, and HEK 293 cells. Cells were incubated with 10 µM of each peptide, or DMSO (vehicle), and then probed by streptavidin-conjugated fluorescein isothiocyanate (FITC). Cells were further stained with ProLong™ Diamond Antifade Mountant with 4′,6-diamidino-2-phenylindole (DAPI) and detected using an epifluorescent EVOS FLoid Cell Imaging Station (ThermoFisher Scientific, Waltham, MA, USA). Green (FITC-peptide); blue (DAPI). (**b**) The corrected total cell fluorescence (CTCF) was determined using ImageJ software, and showed that biotin-GSG-MCA1 and biotin-GSG-MCA2 exhibited significantly higher fluorescence to Mia Paca-2 cells (mean±SEM). As expected, ovarian cancer targeting biotin-GSG-J18 showed significantly higher binding to SKOV-3 cells. A *p*-value of <0.05 was considered statistically significant. **** *p* < 0.0001. GSG, glycine–serine–glycine; DMSO, dimethyl sulfoxide.

Fluorescent microscopy was performed to evaluate binding and cell specificity of peptides biotin-GSG-MCA1 and biotin-GSG-MCA2 to Mia Paca-2 cells, hTERT-HPNE, SKOV-3, LNCaP, and HEK 293 cells. The cells were grown on cell-binding chamber slides and incubated with 10 µM peptide. Bound peptides were probed by streptavidin-FITC and visualized using epifluorescent microscopy. The fluorescent intensity per cell was calculated to quantify peptide binding. The results showed that biotin-GSG-MCA1 and biotin-GSG-MCA2 bound significantly higher (*p* < 0.0001) to MiaPaca-2 cells compared to DMSO (vehicle). Further, peptide binding was found to not be significant to any of the other cell lines (hTERT-HPNE, SKOV-3, LNCaP, and HEK 293), suggesting that MCA1 and MCA2 preferentially bind to Mia Paca-2 cells (Figure 1).

### 3.3. Modified ELISA

**Figure 2:**
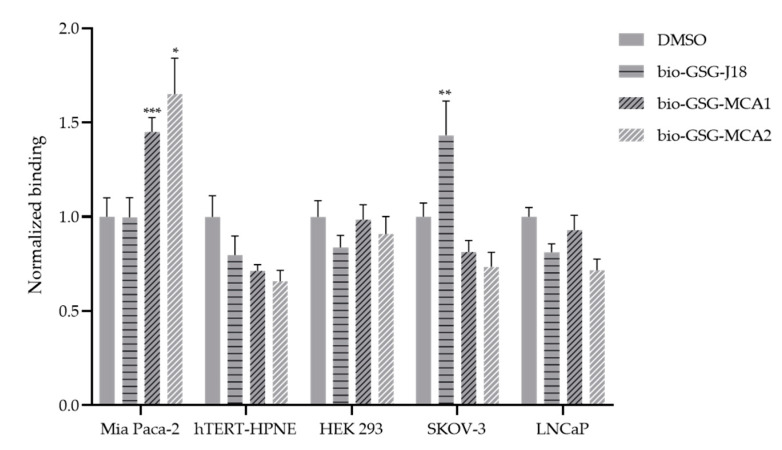
Binding of peptides biotin-GSG-MCA1, biotin-GSG-MCA2, and biotin-GSG-J18 to Mia Paca-2, hTERT-HPNE, SKOV-3, LNCaP, and HEK 293 cells. Cells were grown in appropriate growth medium on 96-well plates. Peptides, MCA1, MCA2, J18 (10 μM), or DMSO were incubated with each cell line for 1 h, then fixed by 10% formalin, and blocked using 10% FBS, 0.3 M glycine, 0.05% Tween-20. Peptide binding was then probed with streptavidin-HRP and detected spectrophotometrically at 405 nm after addition of ABTS using a SpectraMax 250 microplate reader (Molecular Devices, San Jose, CA, USA). Binding of peptides MCA1 and MCA2 to Mia Paca-2 cells was significantly higher compared to J18 and DMSO (mean ± SEM). Binding of J18 to SKOV-3 cells was significantly higher compared to MCA1, MCA2 and DMSO. * *p* < 0.05, ** *p* < 0.01, *** *p* < 0.001.

Peptide binding assays were performed to further determine if peptides MCA1 and MCA2 exhibited significant binding to Mia Paca-2 cells compared to controls (DMSO, and ovarian cancer targeting peptide, J18) [15,16]. A horseradish peroxidase assay was performed to determine cell binding. For each cell line, the binding was normalized to DMSO (vehicle) by dividing the absorbance of each peptide with the absorbance for DMSO (A_peptide_/A_DMSO_). The results showed that peptides biotin-GSG-MCA1 and biotin-GSG-MCA2 bound significantly higher (*p* < 0.05) to Mia Paca-2 cells compared to DMSO and J18. Similarly, modified ELISA assays were performed to determine if peptides MCA1 and MCA2 bound to normal pancreatic cells (hTERT-HPNE), a non-relevant ovarian adenocarcinoma cell line (SKOV-3), a human prostate cancer cell line (LNCaP), as well as human embryonic kidney cells (HEK 293). These results showed that peptides MCA1 and MCA2 exhibited no significant binding to hTERT-HPNE, SKOV-3, LNCaP, or HEK 293 cells, indicating that the peptides may be specific for Mia Paca-2 cells. As validation, peptide J18, which is known to bind specifically to SKOV-3 ovarian cancer cells, showed significantly increased binding (*p* < 0.01) to the ovarian cancer cell line SKOV-3, but not to Mia Paca-2, hTERT-HPNE, LNCaP, or HEK 293 cells (Figure 2).

**Figure 3:**
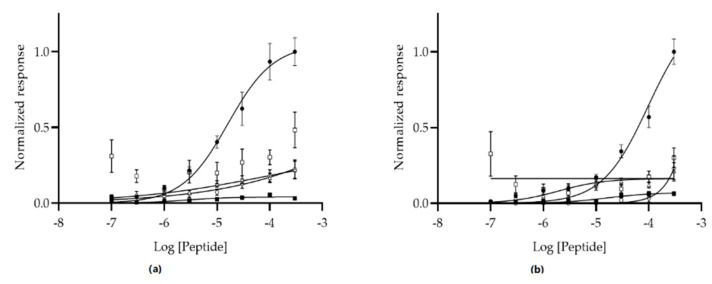
Dose–response study of biotin-GSG-MCA1 and biotin-GSG-MCA2 binding to Mia Paca-2 (•), hTERT-HPNE (□), HEK 293 (Δ), SKOV-3 (▪), and LNCaP (◦) cell lines. Varying concentrations (0.1–300 µM) of peptides were added to the cells for 1 h. Peptides were probed with HRP-conjugated streptavidin and binding was measured spectrophotometrically at 405 nm after addition of ABTS (SpectraMax 250 microplate reader, Molecular Devices, San Jose, CA). Dose–response curves were fitted using GraphPad Prism (vs. 8.3.0). (**a**) The EC_50_ value for biotin-GSG-MCA1 incubated with Mia Paca-2 cells was 16.11 µM (CI [9.69, 26.31 µM]). (**b**) The EC_50_ value for biotin-GSG-MCA2 incubated with Mia Paca-2 cells was 97.01 µM (95% CI [58.64, 166.30 µM].

The peptide binding was further investigated by determining the EC_50_ values using a cell-based dose–response assay. Mia Paca-2, hTERT-HPNE, SKOV-3, LNCaP, or HEK 293 were incubated with 0.1–300 µM biotin-GSG-MCA1, or biotin-GSG-MCA2, for 1 h followed by the measurement of absorbance at 405 nm after addition of streptavidin-HRP and ABTS. For each cell line, the binding was normalized to the absorbance of the highest concentration of peptide (300 µM) for the Mia Paca-2 cells. The results showed that biotin-GSG-MCA1 and biotin-GSG-MCA2 followed a sigmoidal dose response curve, from which it was possible to determine respective EC_50_ values. Peptides biotin-GSG-MCA1 and biotin-GSG-MCA2 exhibited EC_50_ values of 16.11 µM (95% confidence interval; CI [9.69, 26.31 µM]) and 97.01 µM (95% CI [58.64, 166.30 µM]), respectively. The results further showed that it was not possible to model a dose–response curve or determine confidence intervals for the binding of biotin-GSG-MCA1 and biotin-GSG-MCA2 to hTERT-HPNE, SKOV-3, LNCaP, or HEK 293 cells, indicating that the peptides are specific for Mia Paca-2 cells (Figure 3). The EC_50_ value of 97.01 µM for biotin-GSG-MCA2 is approximately 5–10 higher compared to peptides that are successfully used in targeting of various tumors [15,16]. Thus, peptide MCA2 was eliminated from further studies.

**Figure 4:**
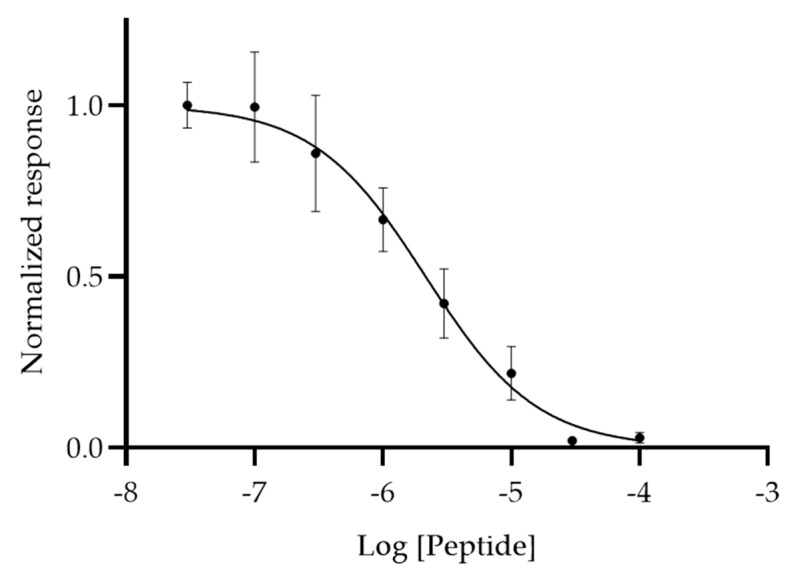
Dose–response study of MCA1 binding to Mia Paca-2 cells. Varying concentrations (0.03–100 μM) of GSG-MCA1 were added to the cells for 15 min, after which 30 μM biotin-GSG-MCA1 was added for 1 h. Peptides were probed with HRP-conjugated streptavidin and binding was measured spectrophotometrically at 405 nm after addition of ABTS (SpectraMax 250 microplate reader, Molecular Devices, San Jose, CA, USA). The dose–response curves were fitted using GraphPad Prism (vs. 8.3.0). The IC_50_ value for MCA1 was 2.15 µM (95% CI [1.28, 3.62 µM]).

A competitive binding assay was utilized for the determination of the IC_50_ values of biotin-GSG-MCA1 for Mia Paca-2 cells. The other cell lines were not employed in this experiment, since only negligible binding was observed for these. The Mia Paca-2 cells were preincubated with 0.03–100 μM GSG-MCA1 for 15 min, and 30 μM biotin-GSG-MCA1 peptide was then added followed by the measurement of absorbance at 405 nm after addition of ABTS. The results showed that biotin-GSG-MCA1 followed a sigmoidal dose–response curve and exhibited an IC_50_ value of 2.15 µM (95% CI [1.28, 3.62 µM]), indicating binding specificity of the peptide to Mia Paca-2 cells (Figure 4).

## 4. Discussion

Pancreatic cancer is currently the fourth leading cause of cancer-related deaths in the United States, and the disease is characterized by an average 5-year survival rate of approximately 3% [1]. The high mortality rate is in part due to asymptomatic disease progression at the early stages, as well as inadequate diagnostic techniques [2,3,4,5,6]. The current “gold standard” for the detection of pancreatic cancer is the measurement of serum CA 19-9. However, low expression levels at the early stages of the disease make detection difficult. Furthermore, expression of CA19-9 has been associated with several other cancer types, including ovarian and breast cancers [7,8,9,10]. In addition, the use of standard imaging techniques for diagnosis, such as computed tomography (CT) and endoscopies, is complicated by the small nature (10–30 mm) of pancreatic tumors [4,5,6]. Thus, it is essential to identify other methods for detecting and diagnosing pancreatic cancer.

Phage display technology may be employed to discover new peptide ligands that target pancreatic cancer and that could be used in detection of the disease [40]. In this study, phage display was utilized to discover peptides that bind to Mia Paca-2 pancreatic cancer cells. In brief, a negative round of selection using a 15-mer fUSE5 phage library was performed against normal pancreatic cells (hTERT-HPNE) to remove unwanted phages from the library. The cleared library was then subjected to four rounds of positive selection against Mia Paca-2 cells. Next-generation sequencing of phages from the negative and fourth positive selection, and Log2 analysis of the most abundant clones, identified two clones, pMCA1 and pMCA2, with a fold change >1.5.

Peptide sequences from the selected phage clones were synthesized separately from the phage virion to determine if peptides MCA1 and MCA2 exhibit specificity for the pancreatic cancer cells. The binding properties of peptides MCA1 and MCA2 were studied using a modified ELISA, as described previously, and fluorescent microscopy [16]. The results showed that peptides MCA1 and MCA2 bound significantly higher to Mia Paca-2 cells (*p* < 0.01) when compared to DMSO. As expected, peptide J18, which is known to bind specifically to SKOV-3 human ovarian cancer cells, failed to bind to Mia Paca-2 cells [17]. These results indicate that MCA1 and MCA2 binding was mediated by these peptides, and not merely by the presence of a peptide. The binding properties of peptides MCA1 and MCA2 were further studied in immortalized pancreatic cells (hTERT-HPNE) and human embryonic kidney cells (HEK 293). The results demonstrated that peptides MCA1 and MCA2 do not bind to the two cell lines, suggesting that MCA1 and MCA2 bind preferentially to Mia Paca-2 cancer cells. In order to investigate if peptides MCA1 and MCA2 bind specifically to pancreatic cancer Mia Paca-2 cells, or to any cancer cells, two additional cancer cell lines, ovarian (SKOV-3) and prostate (LNCaP), were used. The results from both the modified ELISA and fluorescent microscopy demonstrated that MCA1 and MCA2 displayed little binding to either SKOV-3 or LNCaP cells, indicating that both peptides bind preferentially to Mia Paca-2 cells. Thus, the peptides are most likely not binding to antigens that are expressed by several different types of cancer, such as the Thomsen–Friedenreich (TF) antigen, galectin-3, or the epidermal growth factor receptor (EGFR) [21,41,42,43,44]. Thus, the binding pattern of MCA1 and MCA2 suggests that the antigen is specific to pancreatic cancer cells. Previous studies have shown that cancer antigen 19-9 (CA19-9) and mucin 4 (MUC4) are upregulated in pancreatic cancer, and these may be the targets of our selected peptides [4,8,9,45]. Two less studied predictive markers of pancreatic cancer are deoxycytidine kinase (DKC) and human equilibrative nucleoside transporter (hENT1), which have both been found to be overexpressed in the disease [46,47]. Therefore, these proteins may also be likely candidates for the MCA1 and MCA2 target(s).

Peptides MCA1 and MCA2 may have the potential to be used in cancer detection and imaging due to the observed specificity for Mia Paca-2 pancreatic cancer cells. Other peptides that were identified using phage display technology have proved to be useful in targeting disease biomarkers. Peptide J18 was discovered using a two-tier phage display selection to bind to SKOV-3 human ovarian cancer cells and was successfully used in SPECT/CT imaging of xenografted SKOV-3 tumors in mice. The images revealed good tumor uptake of the peptides as well as minimal background binding [17]. Similarly, peptide VINP28 was selected to bind the vascular cellular adhesion molecule-1 (VCAM-1), which is known to be upregulated in atherosclerotic plaques [48]. Numerous other studies have reported the discovery of phage display-derived peptides that effectively detect and image cancer, including breast, prostate, and ovarian malignancies [16,25,49,50]. In this regard, peptides offer many advantages including rapid biodistribution, high tumor penetration, and low immunogenicity [23,24,51]. However, compared to antibodies, first-generation peptides often suffer from relatively low binding affinity. Generally, peptides should exhibit EC_50_ values at least in the low µmolar range to be successfully used in detection and imaging. For example, ovarian cancer-targeting peptides M6 and M9 showed EC_50_ values of 22.9 ± 2.0 μM and 12.2 ± 2.1 μM (mean ± STD), respectively, and were utilized in optical imaging of tumors [16]. Additionally, ovarian-cancer -specific peptides J18 and J24 exhibited EC_50_ values of 22.2 ± 10.6 μM, and 29.0 ± 6.9 μM (mean ± SE), respectively [15]. Here, a dose–response study was conducted to determine the EC_50_ values of MCA 1 and MCA2 against Mia Paca-2, hTERT-HPNE, HEK 293, SKOV-3, and LNCaP cells. The results revealed that biotin-GSG-MCA1 and biotin-GSG-MCA2 exhibited EC_50_ values of 16.11 µM (95% CI [9.69, 26.31 µM]) and 97.01 µM (95% CI [58.64, 166.30 µM]), respectively. Additionally, it was not possible to model a dose–response curve or determine confidence intervals for the binding of the peptides to hTERT-HPNE, SKOV-3, LNCaP, or HEK 293 cells, reflecting the results from the modified ELISA and fluorescent microscopy experiments. Thus, these data further imply that both peptides bind preferentially to pancreatic cancer Mia Paca-2 cells. The EC_50_ value for biotin-GSG-MCA1 is in the lower μmolar range, which has previously been reported to be the upper limit for successful peptide ligands [15,16,52]. Taken together, the EC_50_ values of effective cancer targeting peptides reported in the literature suggest that peptide MCA1 exhibits adequate binding affinity to be employed in the detection and imaging of pancreatic cancer. To the contrary, biotin-GSG-MCA2 exhibited an EC_50_ value in the mid μmolar range and was thus eliminated from further studies.

A competitive dose–response experiment was carried out to determine if MCA1 exhibits specific binding for an antigen expressed by Mia Paca-2 cells. The binding followed a sigmoidal dose–response curve, indicating binding specificity of the peptide to Mia Paca-2 cells. The study revealed an IC_50_ value of 2.15 µM (95% CI [1.28, 3.62 µM]) for MCA1, which is comparable to previously reported phage display-derived peptides. Of these, ovarian-cancer-targeting peptide J18 was reported to exhibit an IC_50_ value of 10.5 ± 1.1 μM (mean±std) and was effectively used in imaging of xenografted ovarian tumors in mice [17]. In addition, peptide MIP that was selected to bind Src homology 3 (SH3) domain of human mixed-lineage kinase 3 (MLK3) displayed an IC_50_ value of 15.8 ± 0.4 μM (mean±std) [53]. Further, a peptide targeting the breast cancer biomarker cysteine-rich intestinal protein 1 (CRIP1) showed an IC_50_ value of 8.8 μM [54]. Taken together, these results indicate that MCA1 exhibits adequate binding specificity for Mia Paca-2 cells, and may, therefore, be employed in detection of pancreatic cancer.

## 5. Conclusions

Phage display technology was used to select peptides that target pancreatic cancer Mia Paca-2 cells and resulted in the identification of two peptides, MCA1 and MCA2. Fluorescent microscopy and modified ELISA experiments demonstrated that both peptides exhibit cell specificity for the Mia Paca-2 cell line. The ligands were further investigated to measure their binding affinities. A dose–response study revealed that MCA1 and MCA2 display EC_50_ values of 16.11 µM (95% CI [9.69, 26.31 µM]) and 97.01 µM (95% CI [58.64, 166.30 µM]), respectively. Based on these results, peptide MCA1 was selected for further studies to determine binding specificity using a competitive dose–response study. From this, the IC_50_ value of MCA1 was found to be 2.15 µM (95% CI [1.28, 3.62 µM]). Both the EC_50_ and IC_50_ values for MCA1 correlate with previously reported phage display-selected peptides that have been successfully employed in cancer targeting [15,16,17,54]. Thus, peptide MCA1 may be used in detection of pancreatic cancer to diagnose the disease.

## Figures and Tables

**Table 1:** Phage display selected clones were identified by next-generation sequencing. The fold change (Log2) was calculated for the most abundant clones. Clones with Log2 > 1.5 were selected for further study.

Peptide	Log2 (pos/neg)
MCA1	6.03
MCA2	3.73
MCA3	−1.86
MCA4	−3.61
MCA5	−2.49
MCA6	−3.98
MCA7	−3.77
MCA8	−3.90
MCA9	−3.97
MCA10	−3.43
